# Lean mass and associated factors in women with PCOS with different phenotypes

**DOI:** 10.1371/journal.pone.0292623

**Published:** 2023-10-05

**Authors:** Tayane Muniz Fighera, Betânia Rodrigues dos Santos, Poli Mara Spritzer

**Affiliations:** 1 Gynecological Endocrinology Unit, Division of Endocrinology, Hospital de Clínicas de Porto Alegre, Porto Alegre, Rio Grande do Sul, Brazil; 2 Postgraduate Program in Endocrinology, Medicine School, Universidade Federal do Rio Grande do Sul, Porto Alegre, Rio Grande do Sul, Brazil; 3 Department of Internal Medicine, Universidade Federal do Rio Grande do Sul, Porto Alegre, Rio Grande do Sul, Brazil; 4 Department of Physiology and Postgraduate Program in Physiology, Universidade Federal do Rio Grande do Sul, Porto Alegre, Rio Grande do Sul, Brazil; University of Mississippi Medical Center, UNITED STATES

## Abstract

Although current evidence suggests increased risk of obesity, insulin resistance, and metabolic alterations in patients with polycystic ovary syndrome (PCOS), especially of a hyperandrogenic phenotype, the impact of each one of these variables on muscle mass remains uncertain. In this case-control study, we evaluated clinical and hormonal characteristics related to lean body mass according to the different PCOS phenotypes. We performed clinical, metabolic, and hormonal assessments and evaluated body compartments by dual-energy X-ray absorptiometry in 133 women of reproductive age. Creatinine served as an indirect marker of lean mass. Median age was 28 (range, 17–37) years. Women with phenotypes A and B (n = 59) had higher body mass index (BMI) and metabolic syndrome prevalence than those with phenotype C (n = 23) and controls (n = 51) (p**<**0.005). Women with phenotypes A and B also had higher Ferriman-Gallwey score (p<0.001), insulin levels (p = 0.006), HOMA-IR (p = 0.008), testosterone (p = 0.008), free androgen index (FAI) (p<0.001), fat mass index (FMI) (p = 0.015), android-to-gynoid fat ratio (p = 0.036), and bone mineral density (BMD) at lumbar spine (p = 0.027) and total femur (p = 0.013) than controls. Median appendicular lean mass index (ALMI) was higher in phenotypes A and B than in controls (7.01 [IQR, 6.33–8.02] vs. 6.69 [IQR, 5.94–7.09], p = 0.024), but it did not differ significantly from that in phenotype C (6.60 [IQR, 6.16–7.22], p = 0.222). Even after adjusting for BMI, ALMI correlated positively with creatinine in women with phenotypes A and B (rho = 0.319, p = 0.023) but not in those with phenotype C (p = 0.238) or controls (p = 0.097). In multivariate linear regression analyses, ALMI was positively associated with insulin, FAI, FMI, and total femur BMD. The present results suggest that fasting insulin, FAI, fat mass, and total femur BMD were positively associated with increased lean mass in women with PCOS phenotypes A and B.

## Introduction

Polycystic ovary syndrome (PCOS) is the most common endocrinopathy in women of reproductive age, with a prevalence ranging from 8% to 13% depending on the criteria used [1]. Based on the Rotterdam criteria, the presence of at least two of hyperandrogenism, ovulatory dysfunction, or PCO on ultrasound allow the identification of four different phenotypes: A (hyperandrogenism, ovulatory dysfunction, and PCO morphology); B (hyperandrogenism and ovulatory dysfunction); C (hyperandrogenism and PCO morphology); and D (ovulatory dysfunction and PCO morphology) [1, 2]. In this context, patients with classic PCOS (phenotypes A and B) have shown a more adverse metabolic profile than normoandrogenic patients, including higher prevalence of metabolic syndrome and higher body mass index (BMI) [3–5]. Whether these phenotypes are indicative of the same overall condition and of the same long-term risks in lean body mass parameters has yet to be clarified [6].

Insulin resistance and central obesity are often present in patients with PCOS, features that are strongly associated with adipose tissue dysfunction [7]. Obesity is clearly related to insulin resistance, but even lean women with PCOS are prone to develop insulin resistance [8, 9], affecting about 45% of women as demonstrated in a previous study by our group [10]. Obesity is also a common finding, with a reported prevalence of 30% to 60%, that affects insulin levels regardless of its effects on insulin sensitivity [6, 11, 12]. Although current evidence suggests increased risk of obesity, insulin resistance, and metabolic alterations in patients with PCOS [13], especially of a hyperandrogenic phenotype, the impact of each one of these variables on muscle mass remains uncertain [14, 15].

The conflicting results concerning lean mass in women with PCOS may stem from the detrimental roles of insulin resistance and inflammation [15–17] as well as from the positive effects of hyperandrogenism and obesity [18–20], which reinforces the importance of an analysis by PCOS phenotypes. A recent systematic review and meta-analysis of 45 studies showed that patients with PCOS have increased total and trunk lean mass, a result mainly attributed to overweight/obesity, but it provided conflicting data concerning appendicular lean mass (ALM) [14]. In the present study, we evaluated clinical, laboratory, and bone mass variables related to ALM index (ALMI) according to the classic and ovulatory PCOS phenotypes, compared with those of control women without PCOS. In addition, we investigated serum creatinine, a widely available parameter in clinical practice, as an indirect marker of lean mass.

## Materials and methods

### Study design and participants

In this case-control study, we analyzed biorepository samples from women of reproductive age living in southern Brazil who had undergone body composition assessment by whole-body dual-energy X-ray absorptiometry (DXA). These women were prospectively recruited and participated in studies conducted at our research center from 2010 to 2017, and the data reported in the present study were analyzed between September 2022 and February 2023. Recruitment was carried out through public advertisement calling for women of reproductive age with excessive hair growth (hirsutism) and irregular menses, and for volunteers without hirsutism and with regular menses [21, 22]. The study population with DXA data comprised 82 patients with PCOS and 51 controls (non-hirsute women with regular ovulatory cycles). Diagnostic investigation was performed for all enrolled participants at the endocrine outpatient clinic of the Hospital de Clínicas de Porto Alegre, state of Rio Grande do Sul, Brazil. Inclusion criteria were BMI < 40 kg/m^2^, age between 17 and 40 years, at least 3 years post-menarche, and no intake of drugs known to interfere with hormone levels for at least 3 months prior to the study. Women with diabetes, thyroid dysfunction, and liver or kidney disease were excluded.

PCOS was defined according to the Rotterdam consensus criteria, being diagnosed in the presence of two of the following three traits: 1) oligo-/amenorrhea and/or chronic anovulation (< 9 cycles/year and/or luteal phase progesterone ≤ 3.8 ng/mL); 2) clinical and/or biochemical hyperandrogenism; and 3) PCO appearance on ultrasound after the exclusion of related disorders such as hypothyroidism, Cushing’s syndrome, non-classical congenital adrenal hyperplasia, and hyperprolactinemia. Patients were classified as having either classic PCOS (phenotypes A and B–biochemical and/or clinical hyperandrogenism and oligo-/amenorrheic cycles, with or without PCO appearance on ultrasound) or ovulatory PCOS (phenotype C–biochemical and/or clinical hyperandrogenism, regular ovulatory cycles, and PCO appearance on ultrasound) [1].

The Research Ethics Committee of Hospital de Clínicas de Porto Alegre approved the study protocol, and each study participant provided written informed consent at the time of recruitment. All data underwent pseudonymization prior to data collection and analysis.

### Study protocol

We performed BMI, DXA body composition, and metabolic and hormonal laboratory measurements in participants from all three groups. Blood pressure was measured twice after a 10-minute rest [21, 22]. Hirsutism was defined as a modified Ferriman-Gallwey score ≥ 8 [23]. Free androgen index (FAI) was calculated as testosterone (nmol/L)/sex hormone–binding globulin (SHBG) (nmol/L) × 100. The Joint Interim Statement criteria were used to define metabolic syndrome [24]. The homeostasis model assessment of insulin resistance index (HOMA-IR) was calculated by multiplying insulin (μIU/mL) by glucose (nmol/L) and dividing this product by 22.5 [25].

### Bone mineral density (BMD) and body composition

All participants underwent a standardized physical examination that included measurement of body weight and height. BMI was calculated by dividing body weight (kg) by the square of the height (m^2^).

Body composition was assessed by whole-body DXA using a Lunar Prodigy Primo device (Encore version 14.10; Radiation Corporation, Madison, WI, USA). The coefficient of variation (CV) was 520 g for fat mass and 610 g for lean mass. Total fat mass and total lean mass were obtained by whole-body DXA measurements and expressed in kg. Fat mass index (FMI) was calculated as the ratio of total fat mass to kg^2^. ALM was obtained by measuring lean mass in the arms and legs and expressed in kg. ALMI was obtained as the ratio of ALM to height^2^. An FMI of 6–9 kg/m^2^ and an ALMI ≥ 5.45 kg/m^2^ were considered normal body fat [26].

BMD was measured at the lumbar spine (L1-L4) and right total femur and expressed in g/cm^2^. In the presence of artifacts, the left femur was scanned. The CV was 0.022 g/cm^2^ for the lumbar spine and 0.033 g/cm^2^ for the femur. Z-scores for BMD were calculated using female age-matched controls from the National Health and Nutrition Examination Survey III (NHANES III) study group, and low bone mass was defined as a Z-score ≤ −2.0 SD for age at either skeletal site. Considering that adiposity influences the accuracy of lumbar spine BMD in overweight individuals, total femur BMD was used in the multivariate linear regression models [27]. DXA quality control was performed daily by the same technician, with variation below 2%.

### Laboratory analyses

Blood samples were drawn from an antecubital vein between 8:00 and 10:00 am, after a 12-hour overnight fast. Samples were obtained between days 2 and 10 of the cycle, or on any day in amenorrheic women.

Total cholesterol, high-density lipoprotein cholesterol, triglycerides, and fasting glucose levels were determined by a colorimetric enzymatic method (Siemens Advia 1650, Deerfield, USA). Low-density lipoprotein cholesterol was estimated indirectly by the Friedewald formula [28]. Total testosterone levels (reference values 0.14–0.76 ng/mL) were measured by chemiluminescence immunoassay (Siemens Advia Centaur XP) with a sensitivity of 0.10 ng/mL and intra- and inter-assay CVs of 3.3% and 7.5%, respectively. Plasma insulin and SHBG levels were measured by chemiluminescence immunoassay (Siemens Advia Centaur XP) with a sensitivity of 0.50 μIU/mL and 0.035 nmol/L, respectively, an intra-assay CV of < 3%, and an inter-assay CV of < 5%. Creatinine levels were determined by the Jaffe reaction without deproteinization. Progesterone levels were determined by electrochemiluminescence immunoassay (CentraLink).

### Statistical analysis

Data were obtained from the analysis of biorepository samples. Considering an error of 5%, the interim analysis showed a power of 80% for the difference found in ALMI between the groups.

The Shapiro-Wilk normality test and descriptive statistics were used to assess the distribution of the data. Results are expressed as mean (SD) or median (IQR). Non-Gaussian variables were log-transformed for statistical analysis purposes and then back-transformed into their original units of measure for reporting purposes. Comparisons between group means were analyzed by one-way analysis of variance (ANOVA). Pearson’s chi-square test (χ2) was applied to test categorical variables. Spearman correlation coefficients were calculated between continuous variables. Univariate and multivariate linear regression analysis models were set up to determine the independent effects of study variables on ALMI (dependent variable). Variables significantly associated with ALMI and with an R^2^ value > 15% in the univariate analysis were included in the multivariate models. Data were analyzed with SPSS for Windows, version 21.0 (SPSS Inc., Chicago, IL, USA), and considered to be significant at p < 0.05.

## Results

One hundred and thirty-three women were studied: 59 with phenotypes A and B, 23 with phenotype C, and 51 controls. Overall median age was 28 (minimum 17; maximum 37) years, and the median BMI was 27.9 (IQR, 24.1–33.7) kg/m^2^.

Table 1 summarizes the clinical profile and metabolic and hormonal variables of women with PCOS and controls. Women with PCOS phenotypes A and B had higher BMI (p = 0.002) and metabolic syndrome prevalence (p<0.001) than those with phenotype C and controls. Women with phenotypes A and B also had higher Ferriman-Gallwey score (p<0.001), systolic (p = 0.001) and diastolic blood pressure (p = 0.002), insulin levels (p = 0.006), HOMA-IR (p = 0.008), testosterone (p = 0.008), FAI (p<0.001), FMI (p = 0.015), and android-to-gynoid fat ratio (p = 0.036) than controls. Median ALMI was higher in women with phenotypes A and B than in controls (7.01 [IQR, 6.33–8.02] vs. 6.69 [IQR, 5.94–7.09], p = 0.024), but it did not differ significantly from that in women with phenotype C (6.60 [IQR, 6.16–7.22], p = 0.222). Regarding bone mass, lumbar spine and total femur BMD were higher in women with phenotypes A and B than in controls (p = 0.028 and p = 0.010, respectively), but similar to those in women with phenotype C (p = 1.000 and p = 0.757, respectively).

**Table 1:** Clinical profile and metabolic and hormonal variables of the PCOS and control groups.

Variable	Phenotypes A and B (n = 59)	Phenotype C (n = 23)	Controls (n = 51)	p
Age, years	26 (20–30) ^a^	29 (22–32) ^ab^	28 (24–32) ^b^	0.015
BMI, kg/m^2^	32.3 (25.9–36.4) ^a^	27.2 (23.9–29.6) ^b^	26.7 (23.0–31.1) ^b^	0.002
BMI ≥ 25 kg/m^2^	45 (77.6%)	15 (71.4%)	28 (59.6%)	0.133
DBP, mm Hg	80 (72–81) ^a^	77 (70–80) ^ab^	70 (67–80) ^b^	0.002
SBP, mm Hg	120 (110–130) ^a^	120 (115–125) ^ab^	110 (105–120) ^b^	0.001
Ferriman-Gallwey score	15.0 (9.5–19.5) ^a^	12.0 (9.0–19.2) ^a^	03 (1.7–5.0) ^b^	<0.001
TT, ng/mL	0.65 (0.48–0.82) ^a^	0.60 (0.44–0.70) ^ab^	0.50 (0.41–0.63) ^b^	0.008
SHBG, nmol/L	28.2 (18.9–42.0) ^a^	29.7 (16.6–44.4) ^a^	35.7 (29.7–53.6) ^b^	<0.001
FAI	2.28 (1.42–4.38) ^a^	1.95 (1.36–2.89) ^a^	1.28 (0.81–2.10) ^b^	<0.001
Progesterone, ng/mL	0.80 (0.59–1.41) ^a^	7.39 (5.77–11.53) ^b^	6.44 (0.80–8.56) ^b^	<0.001
Glucose, mg/dL	87 (82–92)	86 (82–90)	86 (81–92)	0.639
Insulin, μU/mL	16.49 (7.81–22.34) ^a^	15.00 (7.02–20.61) ^ab^	9.05 (6.14–12.36) ^b^	0.006
HOMA-IR	3.52 (1.53–5.22) ^a^	3.40 (1.50–4.33) ^ab^	1.93 (1.34–2.53) ^b^	0.008
Triglycerides, mg/dL	103 (59–155) ^a^	87 (66–133) ^ab^	75 (55–105) ^b^	0.011
Total cholesterol, mg/dL	180 ± 35	177 ± 34	177 ± 27	0.873
HDL, mg/dL	43 (37–52) ^a^	47 (39–55) ^ab^	50 (43–60) ^b^	0.010
LDL, mg/dL	107 (87–136)	98 (88–134)	107 (91–122)	0.583
Metabolic syndrome	21 (35.6%) ^a^	3 (13.0%) ^b^	3 (5.9%) ^b^	<0.001
Creatinine, mg/dL	0.69 ± 0.08	0.68 ± 0.12	0.66 ± 0.08	0.403
Total fat mass, kg	36.05 ± 12.42 ^a^	29.81 ± 7.83 ^ab^	29.06 ± 10.86 ^b^	0.005
FMI, kg/m^2^	14.08 (10.18–17.46) ^a^	11.14 (8.80–12.71) ^ab^	10.54 (7.99–15.20) ^b^	0.015
Total lean mass, kg	40.92 (36.19–47.23) ^a^	39.78 (36.08–42.71) ^ab^	38.07 (35.21–40.82) ^b^	0.010
ALMI, kg/m^2^	7.01 (6.33–8.02) ^a^	6.60 (6.16–7.22) ^ab^	6.69 (5.94–7.09) ^b^	0.020
Lumbar spine BMD, g/cm^2^	1.258 ± 0.120 ^a^	1.249 ± 0.101 ^ab^	1.194 ± 0.136 ^b^	0.027
Z-score	0.7 ± 0.9 ^a^	0.5 ± 0.8 ^ab^	0.1 ± 1.1 ^b^	0.014
Total femur BMD, g/cm^2^	1.109 ± 0.143 ^a^	1.064 ± 0.112 ^ab^	1.017 ± 0.163 ^b^	0.013
Z-score	0.5 ± 1.1^a^	0.0 ± 0.6 ^ab^	−0.1 ± 1.0 ^b^	0.004

Data are expressed as mean ± SD, median (IQR), or percentage. P-value by one-way ANOVA or Pearson’s chi-square test. Different lowercase letters in the same row indicate statistically significant differences. PCOS: polycystic ovary syndrome; BMI: body mass index; DBP: diastolic blood pressure; SBP: systolic blood pressure; TT: total testosterone; SHBG: sex hormone–binding globulin; FAI: free androgen index; HOMA-IR: homeostasis model assessment of insulin resistance index; HDL: high-density lipoprotein cholesterol; LDL: low-density lipoprotein cholesterol; FMI: fat mass index; ALMI: appendicular lean mass index; BMD: bone mineral density.

**Fig 1:**
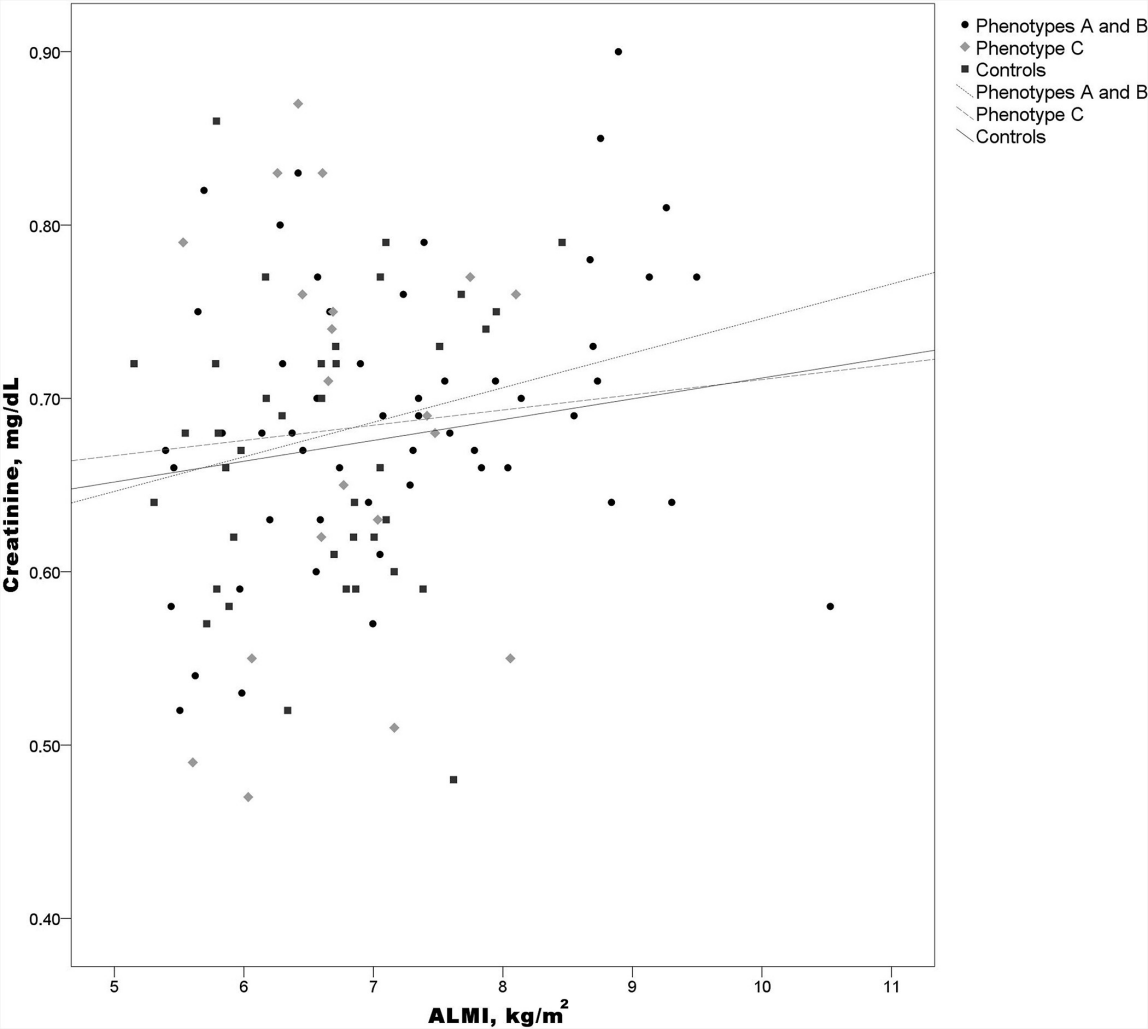
Spearman correlation between ALMI and creatinine in women with phenotypes A and B, phenotype C, and controls. Phenotypes A and B: rho = 0.319, p = 0.023; phenotype C: rho = 0.293, p = 0.238; controls: rho = 0.277, p = 0.097. ALMI, appendicular lean mass index.

Even after adjusting for BMI, ALMI correlated positively with creatinine in women with phenotypes A and B (rho = 0.319, p = 0.023) but not in those with phenotype C (rho = 0.293, p = 0.238) or controls (rho = 0.277, p = 0.097) (Fig 1).

Table 2 shows the variables associated with ALMI in the univariate linear regression analysis: creatinine, insulin, phenotypes A and B, FAI, FMI, BMI, and total femur BMD. Because BMI calculation considers both fat and lean body mass and BMI and ALMI were strongly correlated (rho = 0.958, p<0.001), we did not include BMI in the multivariate models. Likewise, because fasting insulin and FAI were also clearly correlated (rho = 0.548, p<0.001), two multivariate linear regression models were set up, each one using one of these variables separately.

**Table 2:** Univariate linear regression analysis for ALMI.

Independent variable	B (95% CI)	p	R^2^
Age, years	−0.014 (−0.049, –0.021)	0.444	0.3%
Creatinine, mg/dL	2.559 (0.365, 4.752)	0.023	3.8%
Insulin, μU/mL	0.037 (0.024, 0.051)	<0.001	20.0%
Phenotypes A and B	0.430 (0.055, 0.806)	0.025	3.2%
FAI	0.250 (0.148, 0.352)	<0.001	16.6%
FMI, kg/m^2^	0.127 (0.091, 0.162)	0.001	28.1%
BMI, kg/m^2^	0.127 (0.106, 0.149)	<0.001	52.7%
Total femur BMD, g/cm^2^	3.412 (2.261, 4.563)	<0.001	23.3%

ALMI: appendicular lean mass index; FAI: free androgen index; FMI: fat mass index; BMI: body mass index; BMD: bone mineral density.

In the first multivariate linear regression model, fasting insulin, FMI, and total femur BMD were included and all these factors were positively and independently associated with ALMI, explaining 39.3% of the variation in lean mass (Table 3). In the second multivariate linear regression model, using FAI, FMI, and total femur BMD, the positive association between FAI and ALMI was dependent on total femur BMD, and this model explained 36.3% of the variation in ALMI (Table 4).

**Table 3:** Multivariate linear regression analysis for ALMI with fasting insulin.

Variable	Model 1		Model 2		Model 3	
	p<0.001 R^2^ = 18.2%		p<0.001 R^2^ = 28.8%		p<0.001 R^2^ = 39.3%	
	B (95% CI)	p	B (95% CI)	p	B (95% CI)	p
Insulin, μU/mL	0.035 (0.020, 0.049)	<0.001	0.023 (0.008, 0.037)	0.003	0.015 (0.001, 0.029)	0.039
FMI, kg/m^2^	-	-	0.088 (0.044, 0.133)	<0.001	0.075 (0.034, 0.117)	<0.001
Total femur BMD, g/cm^2^	-	-	-	-	2.485 (1.319, 3.652)	<0.001

ALMI: appendicular lean mass index; FMI: fat mass index; BMD: bone mineral density.

**Table 4:** Multivariate linear regression analysis for ALMI with FAI.

Variable	Model 1		Model 2		Model 3	
	p<0.001 R^2^ = 16.5%		p<0.001 R^2^ = 24.6%		p<0.001 R^2^ = 36.3%	
	B (95% CI)	p	B (95% CI)	p	B (95% CI)	p
FAI	0.246 (0.138, 0.354)	<0.001	0.159 (0.044, 0.274)	0.007	0.107 (−0.002, 0.215)	0.054
FMI, kg/m^2^	-	-	0.077 (0.032, 0.122)	0.001	0.063 (0.020, 0.105)	0.004
Total femur BMD, g/cm^2^	-	-	-	-	2.352 (1.271, 3.434)	<0.001

ALMI: appendicular lean mass index; FAI: free androgen index; FMI: fat mass index; BMD: bone mineral density.

## Discussion

In the present study, women with PCOS phenotypes A and B had higher median ALMI than controls, and those with phenotype C showed intermediate results between the other two groups. Women with phenotypes A and B also had a higher prevalence of metabolic and hormonal abnormalities as well as higher lumbar spine and femur BMD than women without PCOS. In addition, fasting insulin, FAI, fat mass, and femoral bone mass were positively associated with ALMI among the study participants. These findings suggest that complex interactions act on the lean body mass composition of women with PCOS, whose clinical presentation is highly heterogeneous.

While the evidence related to fat mass in PCOS seems to be consistent [29–31], data on lean mass remains controversial. A recent meta-analysis showed that women with PCOS had higher lean body mass than controls, but trunk lean mass was comparable between the two groups [14]. The authors stated that the data on ALM remain inconclusive because the studies reported the results inconsistently across upper or lower skeletal regions. The ALMI used in the present study, which is an adjusted ratio of ALM to height, is a validated criterion for assessing lean mass that has shown a consistent correlation with muscle mass throughout life [26].

Adipose tissue secretes acute-phase proteins and other inflammatory mediators, and increased serum pro-inflammatory markers have been reported in women with PCOS compared with healthy controls, leading to chronic low-grade inflammation [32, 33]. Also, as body adiposity increases, there is a concomitant increase in ectopic fat deposition within skeletal muscle, a phenomenon known as myosteatosis [34]. In the present study, despite the higher BMI, patients with PCOS phenotypes A and B had higher ALMI than controls. In fact, fat mass was positively and independently associated with ALMI in the multivariate models that included insulin or androgen levels as well as bone mass. Obesity is known to result in a relative hyperestrogenic state, which may offer protection to skeletal lean mass [35, 36]. Moreover, despite physical function impairment and resistance to anabolic stimuli, weight-bearing muscles may have a greater volume of muscle mass in obese than in lean individuals, which is likely attributable to a larger mechanical stimulus [37]. These findings have been identified in the older population and support a potential *paradox* in which obesity may protect skeletal muscle mass in older age [38].

The anabolic effects of androgens have been demonstrated to increase lean mass in women with hyperandrogenic disorders, including PCOS and congenital adrenal hyperplasia [39]. Hirschberg et al [40] showed an increase in muscle mass and aerobic running time in young healthy women with moderately increased testosterone concentration. Similarly, other studies reported a significant correlation of testosterone and androstenedione levels with lean mass [19] and muscle strength [18] in women with PCOS, although data on PCOS phenotypes are still limited. In the present study, patients with phenotypes A and B, associated with higher androgen levels, had higher ALMI than controls. These findings are in agreement with previous results of our group [20], as we showed increased total and trunk lean mass in women with classic PCOS compared with controls.

Insulin resistance is the hallmark of the metabolic alterations that occur in PCOS, with a higher prevalence in phenotypes A and B [4, 6]. Obesity is a common finding in these patients, and the interaction between body fat excess and insulin resistance seems to be enhanced in patients with PCOS compared with controls [41]. Skeletal muscle is the major site of insulin-mediated glucose uptake, and previous PCOS data have shown an intrinsic defect in insulin receptor signaling as a potential mechanism of insulin resistance [42, 43], which may explain the negative impact on muscle mass reported in some studies [15, 17].

Insulin signaling defects occur after its binding to the receptor and appear to be related to the phosphorylation of serine present in the insulin receptor and insulin receptor substrate 1 (IRS-1) [44, 45]. In patients with PCOS, insulin resistance may be associated with mitogenesis and/or metabolic abnormalities in insulin target tissues [46]. In this respect, insulin modulates protein synthesis and degradation in muscle by both metabolic and mitogenic pathways through the PI3K-Akt/PKB pathway, which regulates the formation of mTOR and GSK3 metabolites [44, 46]. Evidence suggests that the mitogenic actions of insulin on cell growth and differentiation in skeletal muscle are regulated by the MAPK-ERK 1 and 2 pathway, leading to activation of gene expression and protein translation. Indeed, studies of skeletal muscle biopsies from women with PCOS have reported constitutive activation of kinases in the mitogenic MAPK-ERK 1 and 2 pathway, contributing to serine phosphorylation of IRS-1 with consequent inhibition of metabolic signaling in muscle tissue [44–46]. Thus, in women with PCOS and insulin resistance, compensatory hyperinsulinemia through the defects in the signaling cascade could result in activation of mitogenic processes and decrease in metabolic processes. In the present study, both insulin levels and fat mass remained significantly and independently associated with ALMI in the multivariate linear regression model, and with BMD, the model explained 39.3% of the variation in lean mass. Based on these results, an adaptive mechanism with increased muscle mass in response to compensatory hyperinsulinemia could not be ruled out [20, 41].

A limited number of studies have assessed the association between PCOS and bone health, with conflicting results regarding BMD and fractures [47]. Insulin plays a physiological role in osteoblast proliferation, collagen synthesis, and osteocalcin production and inhibits osteoclast activity [48]. However, insulin resistance conditions with compensatory hyperinsulinemia can negatively affect bone mass by suppressing bone turnover as previously demonstrated [49]. Additionally, increased fat mass and high levels of pro-inflammatory cytokines may have a detrimental effect on BMD and induce bone loss [50]. Despite the marked adiposity and insulin resistance reported in classic PCOS, this subgroup has shown increased BMD compared with controls. Sharing a common mechanism of lean mass increase, as previously discussed, androgens are known to stimulate bone growth directly or indirectly via aromatization to estrogen [51]. This established relationship between androgens and bone may explain our multivariate linear regression model in which the association of FAI and ALMI appeared to be dependent on BMD. Indeed, several clinical data have indicated that increased lean mass, as reported in classic PCOS, is associated with a favorable effect on bone mass and reduced fracture risk [52].

Serum creatinine is a classic marker of renal function because the kidney is the main route for creatinine elimination. Creatinine is produced by the non-enzymatic anhydration of creatine in skeletal muscle cells, which contain the highest concentration of creatine in the human body [53]. Therefore, creatinine concentration can be used to estimate muscle mass in different conditions [54–56]. In the present study, ALMI correlated positively with creatinine in women with phenotypes A and B even after adjusting for BMI.

Strengths of our study include having obtained all BMD and body composition data with the same equipment and using strict calibration control procedures, which increases the reliability of the results. Also, data were analyzed according to the different PCOS phenotypes, a key factor in long-term metabolic changes. Limitations are the cross-sectional design, small sample size, serum testosterone assessed by a low-accuracy method, and lack of information about dietary nutrients and physical activity. However, energy intake and habitual physical activity have already been evaluated in a previous study by our group [20].

In conclusion, the results of the present study suggest that women with PCOS phenotypes A and B have increased lean mass, which is associated with fasting insulin, androgens, fat mass, and total femur BMD. Further studies involving participants of different ethnicities as well as addressing dietary and physical activity behaviors are warranted to better understand the crosswalk between lean mass, fat mass, androgens, and insulin levels among women with distinct PCOS phenotypes.

## Supporting information

S1 Checklist STROBE statement—checklist of items that should be included in reports of observational studies. (DOCX)

S1 File (PDF)
