# Supplementary material for: Lean mass and associated factors in women with PCOS with different phenotypes
Source: PLoS One. 2023 Oct 5;18(10):e0292623. doi: 10.1371/journal.pone.0292623 (PMC10553224; doi:10.1371/journal.pone.0292623)
Supplement: S1 File — (PDF) [file pone.0292623.s002.pdf]

Means and standard deviation related to the variables presented in the Table 1.

Variable	Phenotypes A and B (n=59)		Phenotype C (n=23)		Controls (n=51)	
	mean	SD	mean	SD	mean	SD
Age, years	25.2	5.1	26.8	6.1	28.1	4.6
BMI, kg/m^2	31.21	6.53	27.10	3.84	27.40	6.01
DBP, mm Hg	79 ± 9	9	75	8	73	9
SBP, mm Hg	120	11	118	11	112	11
Ferriman-Gallwey score	14	7	13	5	3	4
TT, ng/mL	0.67	0.26	0.60	0.20	0.50	0.16
SHBG, nmol/L	32.8	20.8	30.3	14.1	45.7	21.4
FAI	2.78	1.88	2.40	1.26	1.43	0.99
Progesterone, ng/mL	1.27	2.01	8.17	4.72	6.36	5.42
Glucose, mg/dL	89	2	86	6	87	10
Insulin, µU/mL	18.76	16.15	14.85	8.45	10.73	6.58
HOMA-IR	4.25	3.82	3.18	1.86	2.35	1.62
Triglycerides, mg/dL	116.0	62.5	104.3	47.8	84.5	49.2
Total cholesterol, mg/dL	180	35	177	34	177	27
HDL, mg/dL	45.4	12.6	48.5	11.2	51.8	10.8
LDL, mg/dL	113.1	28.0	107.7	27.7	107.4	21.4
Creatinine, mg/dL	0.69	0.08	0.68	0.12	0.66	0.08
Total fat mass, kg	36.05	12.42	29.81	7.83	29.06	10.86
FMI, kg/m^2	13.52	4.55	11.27	2.97	11.05	4.51
Total lean mass, kg	42.79	7.90	39.67	5.41	38.82	4.93
ALMI, kg/m^2	7.18	1.22	6.68	0.76	6.61	0.82
Lumbar spine BMD, g/cm^2	1.258	0.120	1.249	0.101	1.194	0.136
Z-score	0.7	0.9	0.5	0.8	0.1	1.1
Total femur BMD, g/cm^2	1.109	0.143	1.064	0.112	1.017	0.163
Z-score	0.5	1.1	0.0	0.6	−0.1	1.0

Data are expressed as mean and standard deviation (SD). PCOS: polycystic ovary syndrome; BMI: body mass index; DBP: diastolic blood pressure; SBP: systolic blood pressure; TT: total testosterone; SHBG: sex hormone–binding globulin; FAI: free androgen index; HOMA-IR: homeostasis model assessment of insulin resistance index; HDL: high-density lipoprotein cholesterol; LDL: low-density lipoprotein cholesterol; FMI: fat mass index; ALMI: appendicular lean mass index; BMD: bone mineral density

Individual data of the main variables used in the univariate and multivariate linear regression analysis, presented in Tables 2, 3 and 4, and the correlation (scatterplot), presented in Figure 1.

Number	Age	Total_femur_BMD	Creatinine	BMI	Insulin	Phenotype_AB	FAI	FMI	ALMI
1	29	1.063	0.66	33.68	7.38	0	2.51	16.54	7.05
2	19	0.914	0.65	33.79	12	1	1.86	16.35	7.28
3	23	1.194	0.87	29.02	12.75	0	2.5	14.16	6.42
4	34	0.987	0.49	25.32	30.1	0	999	10.8	5.61
5	33	0.856	0.64	17.14	1.79	0	0.32	3.99	5.31
6	34	1.13	0.75	30.09	18.31	0	1.97	12.98	6.69
7	22	1.088	0.82	23.41	6.4	1	1.09	9.36	5.69
8	17	999	0.6	27.1	22.95	1	4.45	11.73	6.56
9	19	1.23	0.66	33.27	99.67	1	7.69	14.8	8.04
10	30	1.12	0.67	24.55	5.63	1	2.56	10.08	5.4
11	20	1.36	0.59	27.48	6.82	0	1.1	10.51	6.79
12	19	1.084	0.9	34.52	52.5	1	999	15.12	8.89
13	25	1.046	0.68	26.35	9.25	1	0.48	10.35	6.37
14	26	1.042	0.7	33.79	16.29	1	3.03	14.35	8.14
15	33	0.929	0.63	38.28	28.2	0	2.22	19.89	7.1
16	32	0.937	0.57	22.67	6	0	0.52	8.55	5.71
17	34	0.983	0.59	24.03	7.9	0	0.51	10.31	5.79
18	33	0.983	0.67	32.3	999	1	0.92	15.88	7.31
19	37	1.038	0.56	27.73	17.89	0	1.92	999	999
20	30	1.089	0.51	999	20.44	0	3.73	16.51	7.16
21	30	1.044	0.77	43.38	22.54	1	999	22.76	9.5
22	29	1.196	0.69	33.05	7.15	0	999	17.61	6.29
23	29	1.003	0.68	19.56	4.15	1	1.99	5.8	5.83
24	31	1.063	0.71	39.28	999	1	999	17.5	8.73
25	32	1.157	0.63	27.71	12.62	1	1.27	10.12	6.59
26	18	999	0.7	33.06	14.53	1	2.66	14.88	7.35
27	31	0.997	0.69	21.66	7.41	1	0.46	5.38	7.07
28	24	1.373	0.64	41.62	57.2	1	999	19.51	9.3
29	26	1.07	0.73	29.7	28.93	0	1.07	12.4	7.51
30	23	0.86	999	21.56	6.74	0	1.27	6.52	6.42
31	23	1.001	999	22.93	8.45	0	999	8.06	6.22
32	17	999	0.61	33.42	11.11	0	1.5	17.04	6.7
33	33	0.89	0.79	20.14	3.76	0	0.63	3.71	7.1
34	28	1.028	0.72	26.39	6.48	1	2	10.62	6.3
35	26	0.826	0.73	23.16	7.35	0	0.75	7.14	6.71
36	24	0.899	0.58	19.83	6.68	1	1.43	6.09	5.44
37	34	999	0.72	26	9.14	0	0.41	8.79	6.71
38	33	999	0.57	44.26	26.9	0	999	999	999
39	18	999	0.59	24.3	7.11	1	1.55	10.25	5.97
40	23	1.015	999	999	999	0	999	3.61	5.66
41	26	999	999	19.85	999	0	999	999	999
42	29	0.929	0.74	31.96	15	0	2.46	15.45	6.68

43	35	1.329	0.75	26.23	9.05	0	0.98	7.4	7.95
44	27	1.378	0.48	34.31	6.14	0	1.44	15.64	7.62
45	29	0.901	0.68	28.12	6.84	0	2.38	13.74	5.8
46	26	1.099	999	24.74	4.46	1	0.78	9.89	6.37
47	23	1.557	0.85	28.39	14.6	1	1.59	8.84	8.75
48	29	1.005	999	24	25.6	0	5.82	11.1	5.24
49	32	1.267	0.79	35.38	23	1	3.23	18	7.39
50	31	1.056	0.79	21.09	999	0	1.94	6.97	5.53
51	17	999	0.67	34.89	25.6	1	5.82	16.46	7.78
52	28	999	0.65	39.95	17.89	1	1.92	999	999
53	18	1.223	999	28.27	21.77	1	4.41	11.63	6.68
54	33	0.874	0.52	999	5.46	1	0.57	7.97	5.51
55	27	0.873	0.8	20.05	2.6	1	0.61	4.73	6.28
56	19	999	0.68	27.28	11.95	0	3.03	9.71	7.48
57	20	999	0.77	23	9.04	1	1.27	4.64	6.57
58	31	999	0.69	30.78	21.21	1	2.77	13.2	7.35
59	20	1.048	0.63	32.94	16.87	1	1.84	17.35	6.2
60	23	0.956	0.55	24.94	7.3	0	1.11	11.34	6.06
61	33	1.174	0.64	36.59	20.22	1	2.93	16.47	8.84
62	23	1.259	0.78	37.71	19.31	1	2.38	17.42	8.67
63	34	0.593	0.67	23.92	5.22	0	1.88	9.97	5.98
64	29	0.987	0.71	35.38	20.4	1	4.18	16.52	7.94
65	23	1.041	0.62	29.08	14.2	0	1	13.57	6.85
66	31	1.078	999	31.51	9.74	0	2.45	15.52	6.05
67	31	999	999	20.23	4.7	0	1.07	999	999
68	25	1.262	0.77	28.88	28.95	0	4.56	11.13	7.75
69	29	1.023	0.7	18.95	4.1	0	999	3.75	6.17
70	34	1.018	0.83	23.8	2.86	0	1.45	8.66	6.61
71	35	0.898	999	28.91	12.33	0	1.28	13.05	6.08
72	32	1.282	999	32.32	18.69	1	0.39	14.09	7.02
73	28	1.228	0.83	23.5	999	1	999	7.77	6.42
74	31	1.038	999	30.4	10.61	0	3.28	14.09	6.83
75	30	0.76	0.52	22.2	999	0	0.69	6.9	6.34
76	25	0.957	0.6	21.13	10.87	0	1.22	5.17	7.16
77	23	1.275	0.68	37.47	23.6	1	6.27	19.48	7.59
78	20	1.141	0.7	38.66	18.55	1	2.6	20.15	6.56
79	24	0.932	0.72	20.56	7.58	0	0.77	7.05	5.15
80	29	0.971	0.47	26.33	18.93	0	1.34	11.15	6.03
81	30	1.253	0.66	32.52	13.22	1	5.58	13.57	7.83
82	30	0.76	999	999	999	0	0.69	6.9	6.34
83	26	0.891	0.75	21.5	4.51	1	1.37	7.88	5.65
84	29	0.837	0.58	27.91	9.35	0	0.79	12.5	5.89
85	24	1.057	0.68	22.47	6.59	1	1.45	7.21	6.14
86	27	1.082	0.64	25.62	5.31	1	2.76	9.99	6.96
87	22	1.045	0.63	26.13	20.78	0	1.33	8.95	7.03
88	31	1.003	0.62	30.52	27.08	0	5.27	13.97	7.01

89	19	1.144	0.66	28.74	11	1	7.04	11.38	6.74
90	18	999	0.62	27.49	11.44	0	1.48	12.43	6.6
91	20	0.99	0.86	21.89	17.03	0	2.01	8.31	5.79
92	21	0.972	999	26.73	5.74	1	1.56	12.62	5.55
93	35	1.085	0.79	24.79	5.09	0	2.21	7.93	8.46
94	19	1.123	0.57	26.9	28.97	1	4.63	11.55	7
95	31	1.19	0.55	34.52	24.3	0	2.5	13.72	8.06
96	18	999	0.54	24.14	12.39	1	2.25	11.07	5.62
97	28	1.107	0.66	25.29	5.94	0	0.83	10.82	5.86
98	28	1.269	999	39.34	999	0	999	17.22	8.91
99	24	1.213	0.69	37.45	24.42	1	4.77	17.91	8.55
100	17	999	0.81	39.1	41.35	1	4.39	19.14	9.26
101	21	1.14	0.77	24.62	11.16	0	1.33	9.26	6.17
102	23	999	0.61	35.86	13.52	1	1.47	19.06	7.05
103	26	0.983	0.7	27.22	12.49	0	1.14	10.82	6.6
104	26	1.371	0.58	44.36	36.1	1	6.07	18.78	10.53
105	28	1.174	0.67	27.12	23.2	1	1.81	11.06	6.46
106	21	1.192	999	39.52	19.5	1	2.28	19.7	8.01
107	32	0.965	0.65	29.3	17.06	0	4.42	12.45	6.77
108	28	0.951	0.76	33.18	12.75	0	1.47	14.89	7.68
109	25	1.204	0.72	19.54	20.38	0	1.07	5.63	5.78
110	34	1.328	999	25	12.43	0	0.42	10.34	5.98
111	29	0.996	0.62	33.43	9.96	0	1.44	18.32	5.92
112	29	0.966	0.66	37.12	19.95	1	2.2	20.07	5.46
113	28	1.01	0.71	27.84	4.81	0	1.62	12.28	6.65
114	31	1.008	0.72	30.8	6.57	0	2.27	15.89	6.6
115	28	999	0.73	36.3	12.66	1	1.39	16.38	8.7
116	34	1.136	0.53	27.41	17.7	1	0.77	11.9	5.98
117	18	999	0.83	21.95	1.53	0	999	7.14	6.26
118	22	1.082	0.51	999	999	0	999	999	999
119	24	1.128	0.76	33.8	50.71	1	5.12	15.8	7.23
120	32	1.148	0.71	36.93	16.7	1	4.36	17.71	7.55
121	34	0.811	0.68	21.38	8.69	0	0.26	7.42	5.55
122	26	1.078	0.59	38.05	11.94	0	1.05	20.25	7.38
123	20	1.208	0.74	28.08	16.03	0	3.85	10.63	7.87
124	18	1.132	0.69	34.31	999	0	2.56	16.39	7.41
125	28	0.944	999	34.98	12.36	0	1.79	16.59	7.76
126	26	0.978	0.64	24.9	5.04	0	999	8.31	6.85
127	31	0.997	0.77	40.76	10.98	1	3.17	18.59	9.13
128	20	0.933	0.59	26.8	999	0	1.18	10.55	6.86
129	33	1.183	0.76	30.42	5.39	0	2.27	11.53	8.1
130	28	1.07	0.77	26.41	5.93	0	1.35	9	7.05
131	30	0.936	0.75	29.59	17.72	1	6.81	13.67	6.66
132	17	1.263	0.76	22.26	9.82	0	1.32	6.67	6.45
133	19	999	0.72	30.79	12.02	1	2.11	14.13	6.9

BMI: body mass index; FAI: free androgen index; FMI: fat mass index; ALMI: appendicular lean mass index. 999: missing; 0 = no; 1 = yes.
